# Vps4b heterozygous mice do not develop tooth defects that replicate human dentin dysplasia I

**DOI:** 10.1186/s12863-018-0699-3

**Published:** 2019-01-11

**Authors:** Aiqin Hu, Ting Lu, Danna Chen, Jin Huang, Weiwei Feng, Yanjun Li, Dan Guo, Xiangmin Xu, Dong Chen, Fu Xiong

**Affiliations:** 1 Department of Medical Genetics, School of Basic Medical Sciences, Southern Medical University, No.1023, Shatai South Road, Guangzhou, 510515 People’s Republic of China; 2 Department of Stomatology, Nanfang Hospital, Southern Medical University, Guangzhou, 510515 China; 3 Guangdong Provincial Key Laboratory of Single Cell Technology and Application, Guangdong, 510515 China; 4 Department of Stomatology, the First Affiliated Hospital of Zhengzhou University, Zhengzhou, 450052 China

**Keywords:** Vacuolar protein sorting 4B, Dentin dysplasia, *Vps4b*^*+/−*^ mice, Phenotype

## Abstract

**Background:**

Vacuolar protein sorting-associated protein 4B (VPS4B) is a member of the ATP enzyme AAA protein family, and is mainly involved in protein degradation and cell membrane fusion. Recently, a dominant mutation in this gene was identified in human dentin dysplasia type I (DD-I). Herein, we report the generation of *Vps4b* knockout (*Vps4b* KO) mice; however, the homozygous *Vps4b* KO mutation was embryonic lethal at the early stages of embryo development, and we therefore report the results of heterozygous mutant mice.

**Results:**

Mice heterozygous for *Vps4b* did not develop tooth defects replicating human DD-I. Immunohistochemistry showed that gene KO was successful, as there was decreased expression of Vps4b in heterozygous mice; hematoxylin and eosin (H&E) staining also showed that the width of the pre-dentin zone was increased in heterozygous mice, although the arrangement of the odontoblasts was not significantly different from wild-type (WT) mice. However, H&E staining showed no obvious abnormalities in the bones of heterozygous mice. Moreover, stereomicroscopic and X-ray radiography results indicated no abnormal manifestations in teeth or bones. Furthermore, statistical analysis of the volume and density of dentin and enamel, as well as skeletal analysis, including the volume and separation of trabecular bone analyzed by micro-CT, all showed no differences between *Vps4b* heterozygotes and WT mice. In addition, there also were no significant differences in bone or cartilage mineralization as evaluated by Alcian blue–Alizarin red staining.

**Conclusions:**

The heterozygous *Vps4b* KO mice do not develop tooth defects that replicate human DD-I and this is likely to be due to differences in tooth development between the two species. Consequently, further studies are needed to determine whether mice are an appropriate animal model for human tooth diseases.

## Background

Vacuolar protein sorting 4 (VPS4) is one of the major members of the ATP enzyme AAA protein family and is known to promote protein degradation and cell membrane fusion [1–3]. In humans, two homologues, *VPS4A* and *VPS4B*, both of which share 80% homology, have been identified [4, 5]. The gene encoding *VPS4A* is located on chromosome 16, while the gene encoding *VPS4B* is located on chromosome 18. Functional studies show that the two genes are associated with the endosomal compartment and are involved in intracellular protein transport [3, 6]. Similarly, the mouse also has two homologues: *Vps4a* and *Vps4b*. The mouse *Vps4a* gene has been mapped to a region of chromosome 8D that shares homology of synteny with human chromosome 16q22, while the mouse *Vps4b* gene has been mapped to a region of chromosome 1E3 that shares homology of synteny with human chromosome 18q21-q22 [7].

Recent studies have found that *VPS4B* primarily participates in lysosomal degradation pathways, intracellular protein transport, viral body budding, and regulation of the different stages of cell division [8, 9]. Previous work from our lab indicated that the *VPS4B* gene was one of the pathogenic genes associated with DD-I, which regulates tooth development via interaction with Wnt/β-catenin canonical signaling [10]. A splicing mutation, IVS7 + 46C > G, genetically linked to DD-I in an extended Chinese family, was identified in VPS4B, and has been proven to cause DD-I in a loss-of-function manner. In addition, our investigation of tooth development in zebrafish or in vitro revealed a function for the *VPS4B* gene in tooth development [10, 11].

In this study, we carried out further studies on the effects of *VPS4B* on tooth development and determined the physiological functions of *VPS4B*. We generated a *Vps4b* knockout (KO) mouse model and analyzed the phenotype of teeth and bones from these mice. The results indicate that heterozygous KO mice do not develop tooth defects that replicate human DD-I.

## Methods

### Transgenic mice

All four *Vps4b* KO mice were heterozygous (HET) (*Vps4b*^+/−^) and produced both wild-type (WT) (*Vps4b*^+/+^) and homozygous mutant (*Vps4b*^−/−^) offspring. The mouse strain is C57BL/6 J from the United States. These mice with gene knockout were provided by Nanjing University-Nanjing Biomedical Research Institute. Mice were bred in SPF - grade barrier environments with the SOP regulations of the barrier facility. The temperature and humidity of laboratory were kept at 23 ± 2 °C and 55 ± 5%. The basket and bedding were replaced every twice week. The 12 h/12 h alternating light/dark illumination method were used. The above-mentioned materials were sterilized with high temperature of 121 °C for 30 min. The mice we used were 1, 3, 6, and 9-month-old from birth. They were euthanized using compressed 5% CO2 gas produced by the delivery systems of Shanghai yuyan science instrument company. The dosage was calculated according to the weight of mice. About 70 days after birth, sexually mature male and female mice were caged in a 1:1 ratio to reproduce. Two groups of comparison are involved. For all experiments, significance between groups was calculated using Student’s t-test. Each group contains at least three mice. Each experiment was repeated at least three times, and a consistent result was accepted [12].

**Fig. 1:**
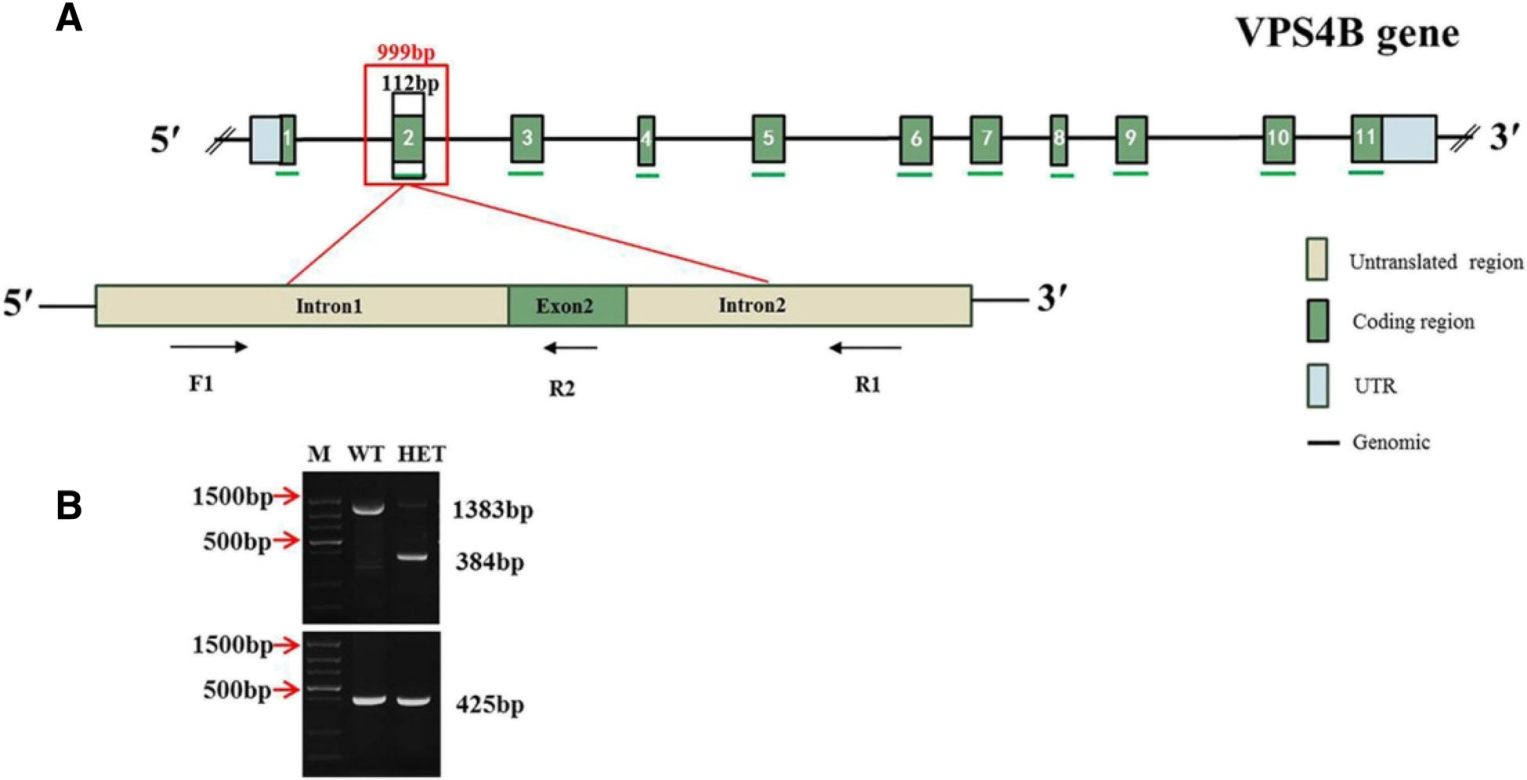
Mouse *Vps4b* gene and PCR primers. **a** Schematics showing the composition of the mouse *Vps4b* gene and the location of PCR primers. The red box depicts the sequence deleted in the transgenic mice. The black box depicts a sequence of exon 2. F1, 5′-TGCTTTAGGCAAAGCAGCAC-3′. R1, 5′-GCTGAAACTGGAGGGTTAGG-3′. R2, 5′-TTTCTGTGTGAGGGAGGCAA-3′. **b** Identification results of the mouse genotypes. WT = wild type mice; HET = heterozygous mutant mice

Polymerase chain reaction (PCR) primers were used to detect the 999 base pair (bp) deletion sequence and are shown in Fig. 1a. Theoretically, if the mouse was WT, the PCR product was 1383 bp, and if the mouse was a homozygous mutant, the corresponding PCR product was 384 bp. Finally, if the mouse was HET, two PCR products were generated, one of each length. However, in actual experiments, HET mice contained the same primer binding sites for each product and therefore the products competed with one another; consequently the knockout alleles were easier to expand than the WT alleles. A primer, reverse2 (R2), inside the deleted sequence was designed to avoid identification errors, and F1 / R2 were used for another PCR reaction. The WT alleles were still present with a 425 bp band while the homozygous mutant contained no band. Using two pairs of primers, we identified mice as WT if they generated a 1383 bp and a 425 bp PCR product; as HET if they generated a 384 bp and 425 bp PCR product; and as homozygous in they generated only a 384 bp PCR product. However, as the homozygous allele was embryonic lethal, only WT and HET mice were generated (Fig. 1b).

### Hematoxylin & eosin (H&E) and immunohistochemical (IHC) staining

The mandibular muscle and gingival tissue were dissected from both WT and HET mice at 1, 3, and 6 months of age. H&E and IHC analysis were performed as previously described [13]. The stained sections were observed and photographed under an inverted fluorescence microscope (Olympus company, Tokyo, Japan). H&E staining was used to observe whether dental pulp cavities were blocked; to identify whether the thickness of the dentin was normal; and to determine the arrangement of odontoblasts. A VPS4B antibody (C-13; Santa Cruz Biotechnology, Santa Cruz, CA, USA) was used in IHC analyses to observe the expression of VPS4B in mouse teeth.

### Plain X-ray radiography and stereomicroscopy

Both WT and HET mice at 3 and 9 months of age, with similar weights, were examined using whole body X-ray imaging to identify any abnormalities in the thickness and mineralization of the skull, ribs, spine, or coccyx. We also employed microcomputed tomography (micro-CT) to observe the teeth and femurs, including the pulp cavity and the ultrastructure of trabecular bone. Mice were also examined under a stereomicroscope for pit depth, occlusal fissures in the teeth, and dental crown height.

### Skeletal Alcian blue–alizarin red staining

Mice were sacrificed and the skin, muscle and visceral tissue were removed. Skeletal specimens were fixed in 95% ethanol for 3 days, then immersed in acetone for 48 h to remove fat. Specimens were then soaked in freshly-prepared staining solution to stain the whole skeletons. The staining time was dependent on the age of the mice. Fetal and juvenile specimens were treated for 1 day, while adult specimens were treated for 3–7 days. Skeletal specimens were then immersed in 1% KOH solution for 24–48 h and transferred to a series of glycerol solutions with 1% KOH solution (volume ratio of 1:4, 1:1, and 4:1, respectively) until the surrounding tissues were completely removed. After bone tissue had become transparent, specimens were immersed in glycerol for long-term preservation. Mineralized bone tissues stained red, while cartilage stained blue.

### Microcomputed tomography (micro-CT)

Procedures for the preparation of mandible samples and micro-CT were as previously described [13]. Briefly, 3-, 6-, and 9-month-old mice were euthanized using compressed 5% CO_2_ gas and their mandibles and femurs were removed and fixed overnight with 4% buffer-saturated paraformaldehyde. Bones were scanned using a Scanco80 (μ80) system (Scanco Medical AG, Bassersdorf, Switzerland). The instrumental isotropic resolution was 10 μm and the iso-surface was reconstructed using two-dimensional raw data using MicroView analysis software (GE Healthcare, Little Chalfont, UK). The image analysis method was based on Hounsfield units (2800 units) and region-grow algorithms to segment image data defining separate anatomical structures. Images were reconstructed with Mimics® 14.0 (Materialise, Leuven, Belgium) software using a global threshold of 1400 Hounsfield units.

### Scanning electron microscopy (SEM)

Mice at 3 and 6 months of age were decapitated to separate the mandibles. Tissues were dissected from mandibles and were placed in 4% paraformaldehyde for 24 h. The tissue was dehydrated using increasing alcohol concentrations (70, 80, 90, and 100%) for 1 week, then soaked in different resin gradients, and finally embedded in the resin. Finally, the tissue was cut with a hard tissue slicer and ground down to fine powder. The jelly samples were sprayed and examined with a Hitachi S3400 N SEM (Tokyo, Japan) to compare differences in the dentin tubule arrangement and density between the different genotypes.

### Statistical analysis

For all experiments, significance between groups was calculated using Student’s t-test. The quantified results are shown as the mean ± SD; *P* < 0.05 was considered to be statistically significant.

## Results

### Effects of heterozygous expression of the *VPS4B* gene on teeth

**Fig. 2:**
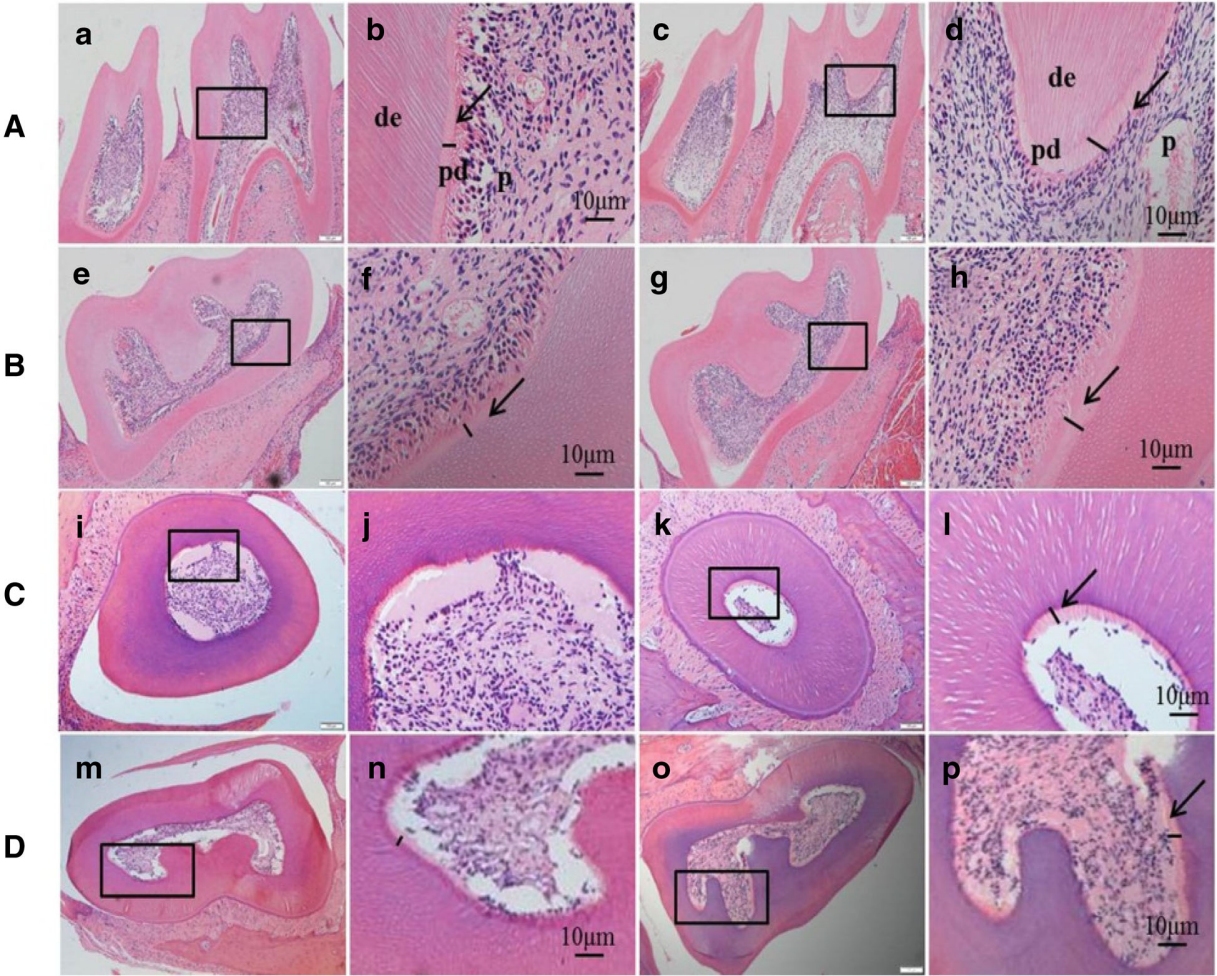
Histopathological analysis of mandibular first molars of mice with increased pre-dentin width. Histopathological analysis of mandibular first molars in 1 (**A**, **B**), 3 (**C**), and 6 (**D**) month-old mice. Compared with WT mice (a, e, i, and m), the pre-dentin width was reduced and the mineralized dentin width was increased in heterozygous mice (c, g, k, and o). **A** Longitudinal sections of a representative 1-month-old mouse tooth. **B**, **C**, and **D** Cross-sections of representative 1-, 3-, and 6-month-old mouse teeth. High-power magnification (b, d, f, h, j, l, n, and p) from the area inside the black box in low-magnification images (a, c, e, g, i, k, m, and o). The black arrows refer to the inner pre-dentin. Abbreviations: de, dentin; p, pulp; pd., pre-dentin

Pre-dentin is a layer of dentin that forms prior to dentin mineralization. Pre-dentin attaches to the pulp cavity; and its thickness represents the mineralization rate of dentin [12]. In other words, the thicker the pre-dentin layer, the slower the mineralization. The results of H&E staining of teeth isolated from 1-, 3-, and 6-month-old mice indicated that the width of the pre-dentin layer was increased, but the arrangement of the odontoblasts showed no significant differences between HET mice and WT mice; none of the HET or WT mice displayed pulp cavity obstructions (Fig. 2).

**Fig. 3:**
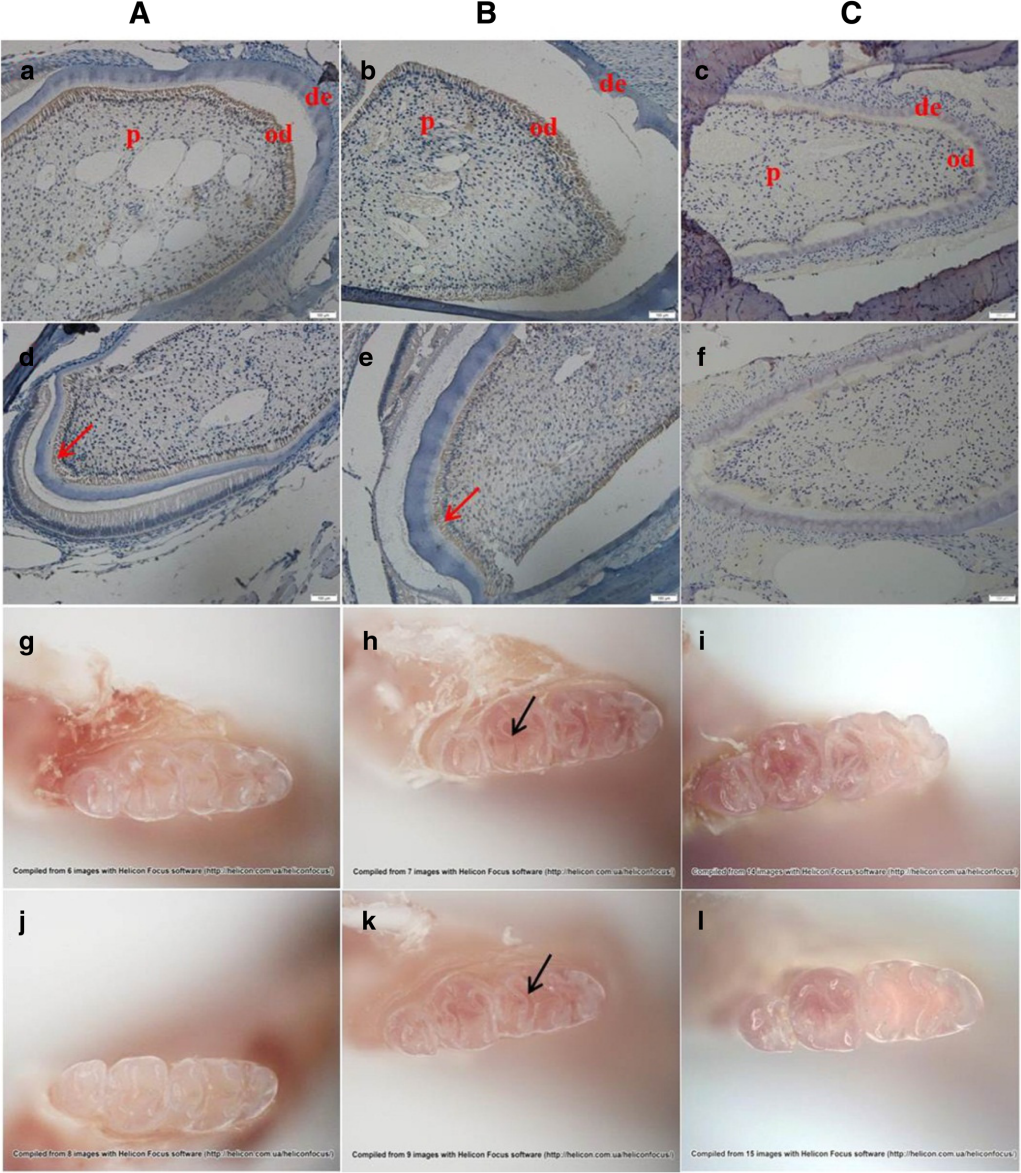
Expression of VPS4B in mouse teeth and stereomicroscopy of mouse teeth. **A**, **B**, and **C** Representative 1-, 3-, and, 6-month-old mouse teeth, respectively. Strong VPS4B signals (*brown*) were observed in the odontoblasts of WT mice (a, b, and c), whereas heterozygous mice (d, e, and f) displayed markedly reduced signals. The red arrows indicate the odontoblast cell layer and the black arrow indicates the pits and fissures of teeth at the occlusal surface. Abbreviations: de, dentin; p, pulp; od, odontoblast (a, b, c, g, h, and i are WT mice; d, e, f, j, k, and l are HET mutant mice)

Dentin is secreted by the odontoblast layer, a single layer of cells close to the exterior of the pulp cavity [14]. The results of immunohistochemical staining suggested that the expression of VPS4B in HET mice was lower than in WT mice, and was specific to the odontoblast cell layer (Fig. 3a-f). As all the mice aged, their teeth became worn and the pits and fissures in the occlusal surface of the teeth became shallow. However stereomicroscopy found no significant difference in the appearance of the teeth or the wear of the occlusal surface between WT and HET mice (Fig. 3g-l).

**Fig. 4:**
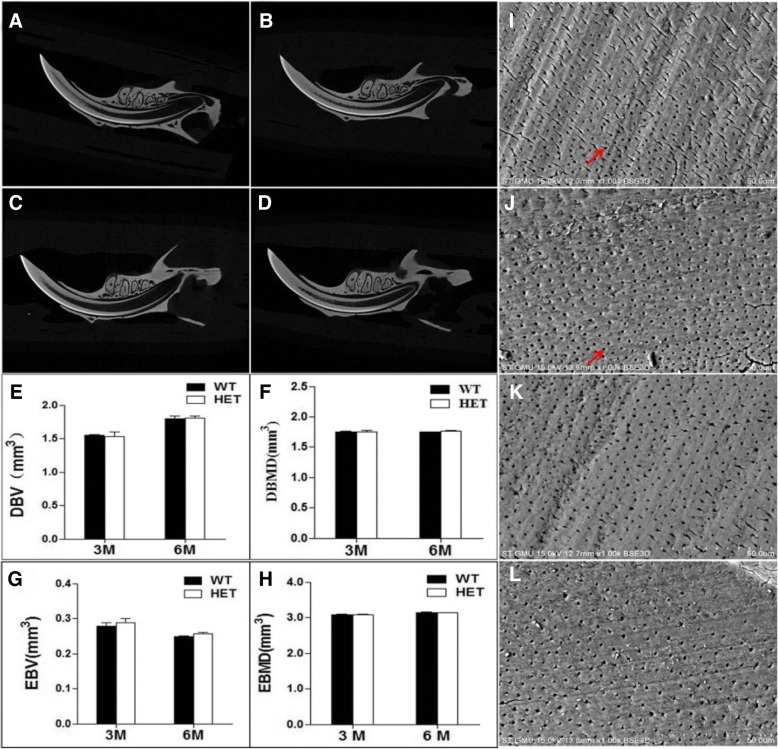
Statistical results of microcomputed tomography of mouse teeth and dentinal tubular structures of molar dentin. **a**, **b** Representative microcomputed tomography of a 3-month-old mouse tooth. **c**, **d** Representative microcomputed tomography of a 6-month-old mouse tooth. **e**, **f**, **g**, and **h** Statistical analysis of DBV, DBMD, EBV, and EBMD on microcomputed tomography of mouse teeth. There were no significant differences between WT mice and HET mice. **i**, **j** Scanning electron microscopy of molar dentin in 3-month-old mice. **k**, **l** Scanning electron microscopy of molar dentin in 6-month-old mice. The red arrows indicate the dentinal tubules. **a**, **c**, **i**, and **k** are WT mice; **b**, **d**, **j**, and **l** are HET mice. Abbreviations: DBV, dentin bone volume; DBMD, dentin bone mineral density; EBV, enamel bone volume; EBMD, enamel bone mineral density

Micro-CT was used to examine the tooth pulp cavity; no significant differences were found between the two genotypes (Fig. 4a-d). We also analyzed the volume and density of dentin and enamel of three photographed teeth on one mandible of 3- and 6-month-old mice. Specifically, the dentin and enamel ranges of the three teeth were drawn to calculate the volume and density of each, with results indicating no significant differences between WT and HET mice. The volume of dentin increased with increasing age, while that of enamel decreased with increasing age, probably due to tooth degeneration. Nevertheless, the mineralization amount of dentin and enamel remained constant (Fig. 4e-h).

We also examined the microscopic structure of teeth from 3- and 6-month-old mice by SEM, to determine the size and sparseness of dentin tubules in dentin cross sections. Both WT and HET mice had dentinal tubules that were normal and uniform in tubular structure (Fig. 4i-l).

**Fig. 5:**
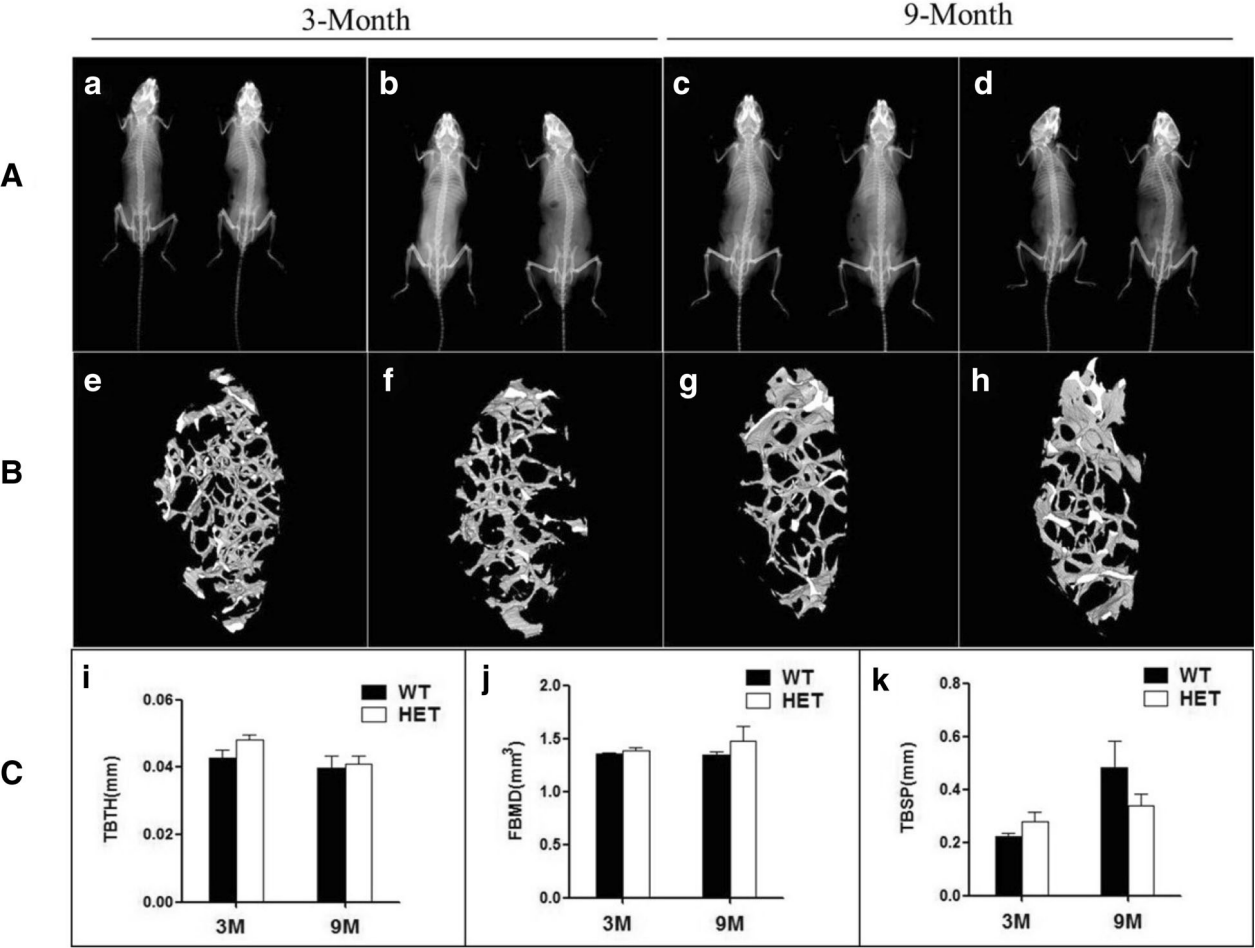
Representative radiographs of mouse skeletal specimens and statistical analysis of trabecular bones analyzed by microcomputed tomography. **A** X-ray analysis of WT and HET mouse skeletal specimens. Two age groups (3- and 9-month-old mice) were analyzed for each genotype; WT mice are shown on the left side of each group; HET mice are shown on the right side. **B** Representative micro-CT 3-dimensional images of trabecular bones of WT mice (e, g) and HET mice (f, h). **C** Statistical analysis of microcomputed tomography of mouse trabecular bones using TBTH, FBMD, and TBSP. Abbreviations: TBTH, trabecular thickness; FBMD, femoral bone mineral density; TBSP, trabecular separation

We investigated the mineralization degree and morphological changes in the ribs, coccyx, and femurs of 3- and 9-month-old mice (Fig. 5A). Micro-CT was also used to observe the ultrastructure of the femoral trabecular bones (Fig. 5B); no significant differences were found in any of the above features between WT and HET mice.

### Effects of the *Vps4B* gene on skeletal structure

**Fig. 6:**
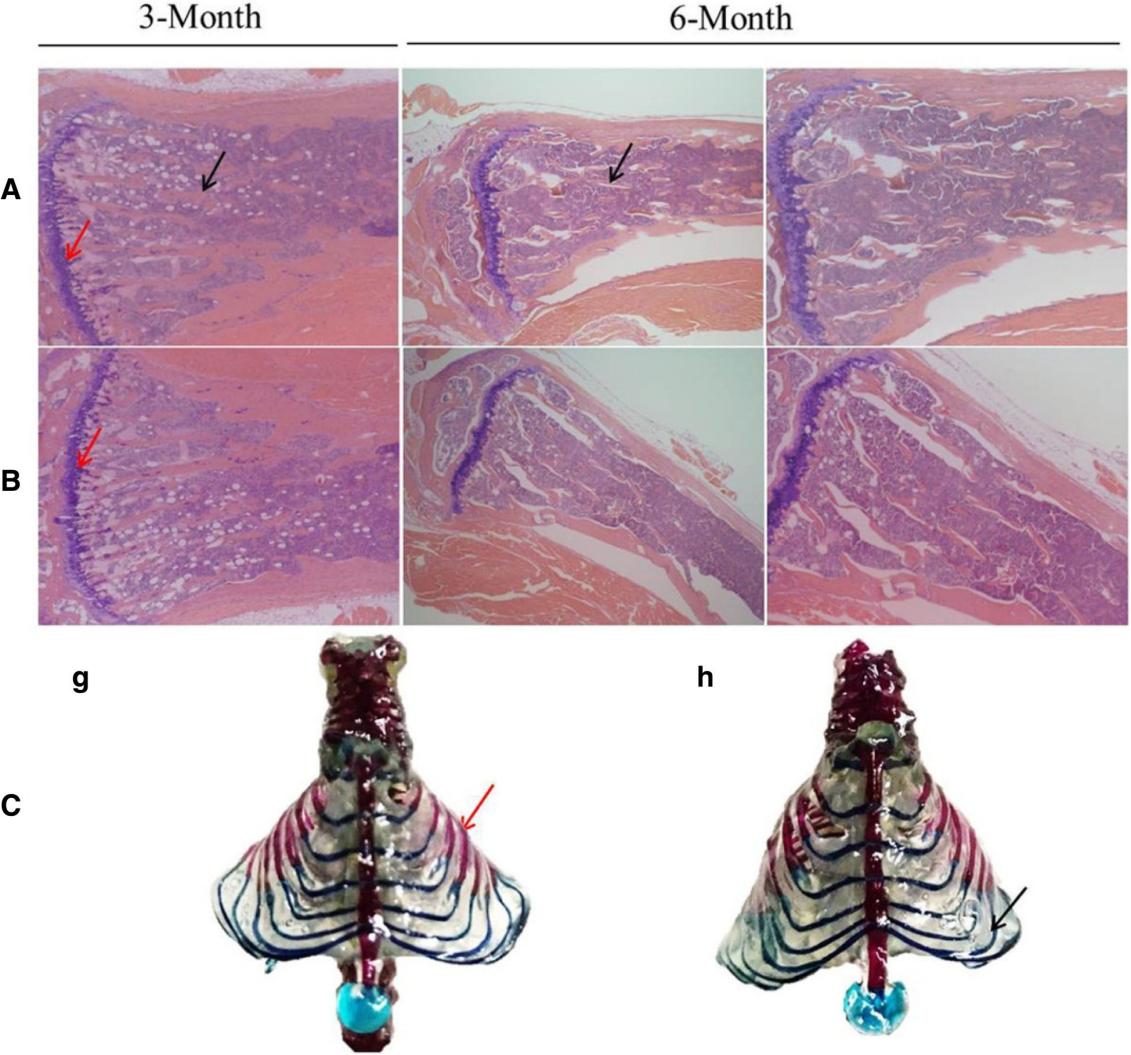
Histological analyses of femoral and skeletal staining. **a**, **b** Representative H&E staining of wild type (WT) (**a**) and HET (**b**) mouse femurs. The black arrows indicate trabecular bone; red arrows indicate growth plates. **c** Alcian blue–Alizarin red staining of skeletal specimens from 6-month-old WT mice (g) and HET mice (h). Black arrows refer to the cartilage and red arrows refer to the mineralized bone tissue

H&E staining of mouse femurs was performed to observe abnormal changes in the trabecular bone (Fig. 6a, b) as bone and dentin are similar in terms of composition and mineralization [15]. As the distance of the trabecular bone from the growth plate is directly related to its mineralization [16], the growth plate is taken as a reference point to observe the trabecular bone density.

We applied Alcian blue–Alizarin red staining of skeletal specimens to analyze the differences with respect to bones and cartilage between the different mice. No differences were found between the two genotypes *(*Fig. 6c)*.*

## Discussion

DD-I (OMIM: 125400) is a rare autosomal dominant dental dysplasia with a penetrance of less than 100% and an incidence of 1/10,000 [17]. Clinical examination shows that the shape and color of teeth are normal, but dentin formation defects and medullary occlusion are often accompanied by a transmission shadow at the root apex in X-rays. Ballschmiede first described the disease in 1920, when he found that seven children from the same family had short, blunt tooth roots and premature tooth loss; a term he named conditional “non root teeth”. Subsequently, mutations in the *SMOC2* gene*, SSHU2* gene and *VPS4B* gene were found to be associated with DD-I [13, 18].

In this study, we compared HET mice to WT mice for differences in teeth and bone, as only HET mice with partial *Vps4b* KO were obtained. Although the expression of VPS4B was reduced and the pre-dentin zone was increased in HET mice compared with WT mice, we did not find any differences in H&E staining of bones, in microscopic results, or in X-ray results. We also used micro-CT to analyze changes in tooth mineralization and volume, as well as volume and density of trabecular bone, while SEM was used to analyze changes in dentinal tubules in the teeth of the two groups. However, no differences were found between HET and WT mice.

Differences in the phenotype caused by disruption of *VPS4B* in human and mouse might be explained by several mechanisms: (1) The different tooth development patterns. It is already known that mouse teeth are constantly growing, a phenomenon which continues throughout life [5]; however, humans have two sets of teeth: deciduous teeth and permanent teeth. At the time of tooth emergence, the root is not fully formed, the medullary cavity is large, and the apical hole is flared open. The apical part is only completely formed at 2–3 years after tooth emergence. (2) The different molecular mechanisms regulating tooth development. Tooth development is a long and complex biological process and is closely related to the migration and directional differentiation of nerve cells [19–21]. There are four molecular signaling families which regulate tooth development, including: bone morphogenetic proteins (BMPs), fibroblast growth factors (FGFs), hedgehog protein family (Hhs) and Wnt signaling [22–24]. Subtle differences in molecular regulation still exist between human and mouse tooth development. For example, FGF8, an important member of the FGF family, exists as four isoforms in the human body (FGF8a, FGF8b, FGF8e and FGF8f) [25]; however, there are eight isoforms in the mouse, named FGF8a–h [26]. Moreover, FGF8 is always expressed in the development of human teeth, while in mouse tooth development, it is initially expressed in the epithelium at the predetermined position before the development of teeth, until the early bud stage, after which it is no longer expressed [26]. In addition, *FRZB* is a key factor in the canonical Wnt signaling pathway for tooth development, which is expressed in the inner enamel epithelium in the human tooth germ family, while *Frzb* was not detected in the mouse tooth germ of El 15.5 [27]. (3) The differences in the genomes between human *VPS4B* and mouse *Vps4b*. Of the 96% of mouse genome sequences studied, up to 99% can be found in humans. The mouse *Vps4b* gene is known to contain six transcripts, among which only *Vps4b*-001 and *Vps4b*-002 transcripts encode translated proteins, and the amino acid sequences of *Vps4b*-001 and *Vps4b*-002 encoding regions are completely consistent, but only *Vps4b*-001 contains RefSeq sequences. By comparing the amino acid sequence of human *VPS4B* protein and mouse *Vps4b*-001 protein, we found that the similarity between them was up to 95%. Consequently we originally wanted to create a mouse that was consistent with the mutation (IVS7 + 46C > G) in the human *VPS4B* gene, but unfortunately, there was no such site in the mouse genome. Therefore we could not replicate the same mutation in the transgenic mouse. Instead, KO mice were constructed by knocking out 999 bp of gDNA from the *Vps4b* gene. (4) The phenotypic changes in heterozygous mice might not be obvious. Because the homozygous allele was embryonic lethal, we were only able to generate *Vps4b* WT and heterozygous mice. Previous studies have reported that dentin matrix protein 1 (DMP1) and dentin sialophosphoprotein (DSPP) play crucial roles in dentin formation. Sreenath et al. deleted the entire DSPP coding region in embryonic stem cells and generated *Dspp*^*−*/**−**^ mice [12, 28]. These null mice exhibited an enlarged pulp cavity, widened pre-dentin zone, decreased dentin width, and high incidence of pulp exposures, similar to type III dentinogenesis imperfecta (DGI-III). However, these differences were only found in homozygous mutants but not in heterozygous mutants. Similarly, it has been reported that similar defects were present in *Dmp1*^−/−^ mice; however, there was no mention of the dental phenotype of heterozygous mice [29, 30]. We speculate the heterozygous mutations in dentin formation-related genes are not sufficient to cause defects in the tooth phenotype in mice.

## Conclusion

We speculate that this result is due to the fact that there are significant differences in tooth formation between mouse and human. In addition, it may be that phenotypic changes of the teeth of heterozygous mice are not obvious, and because we only obtained heterozygous mice, we were not able to see any significant differences in such phenotypic changes. Further investigation into the mechanisms of our experimental findings need to be carried out to determine whether mice are a suitable animal model to investigate human tooth diseases.

## Abbreviations

DD-I: Dentin dysplasia type I
DGI-III: Type III dentinogenesis imperfect
DMP1: Dentin matrix protein 1
DSPP: Dentin sialophosphoprotein
H&E: Hematoxylin and eosin
HET: Heterozygous
IHC: Immunohistochemistry
KO: Knockout
Micro-CT: Microcomputed tomography
PCR: Polymerase chain reaction
SEM: Scanning electron microscopy
VPS4B: Vacuolar protein sorting-associated protein 4B
WT: Wild-type
